# Whole-Genome Sequencing Reveals the Presence of the *bla*_CTX-M-65_ Gene in Extended-Spectrum β-Lactamase-Producing and Multi-Drug-Resistant Clones of *Salmonella* Serovar Infantis Isolated from Broiler Chicken Environments in the Galapagos Islands

**DOI:** 10.3390/antibiotics10030267

**Published:** 2021-03-05

**Authors:** Elton Burnett, Maria Ishida, Sofia de Janon, Sohail Naushad, Marc-Olivier Duceppe, Ruimin Gao, Armando Jardim, Jessica C. Chen, Kaitlin A. Tagg, Dele Ogunremi, Christian Vinueza-Burgos

**Affiliations:** 1 Institute of Parasitology, McGill University, Montreal, QC H9X 3V9, Canada; armando.jardim@mcgill.ca; 2 Division of Food Laboratory, New York State Department of Agriculture and Markets, Albany, NY 12206, USA; Maria.Ishida@agriculture.ny.gov; 3 Unidad de investigación de Enfermedades Transmitidas por Alimentos y Resistencia a los Antimicrobianos (UNIETAR), Facultad de Medicina Veterinaria y Zootecnia, Universidad Central del Ecuador, Quito 170103, Ecuador; dsdejanon@uce.edu.ec; 4 Ottawa Laboratory Fallowfield, Canadian Food Inspection Agency, Ottawa, ON K2J 4S1, Canada; Sohail.Naushad@canada.ca (S.N.); marc-olivier.duceppe@canada.ca (M.-O.D.); Ruimin.Gao@canada.ca (R.G.); 5 Division of Foodborne, Waterborne and Environmental Diseases, Centers for Disease Control and Prevention, Atlanta, GA 30329, USA; lly3@cdc.gov (J.C.C.); nnp2@cdc.gov (K.A.T.); 6 Weems Design Studio Inc., Decatur, GA 30032, USA

**Keywords:** *Salmonella* Infantis, multi-drug resistance, Galapagos, extended-spectrum β-lactamase

## Abstract

*Salmonella* Infantis, a common contaminant of poultry products, is known to harbor mobile genetic elements that confer multi-drug resistance (MDR) and have been detected in many continents. Here, we report four MDR *S*. *Infantis* strains recovered from poultry house environments in Santa Cruz Island of the Galapagos showing extended-spectrum β-lactamase (ESBL) resistance and reduced fluoroquinolone susceptibility. Whole-genome sequencing (WGS) revealed the presence of the ESBL-conferring *bla*_CTX-M-65_ gene in an IncFIB-like plasmid in three *S*. Infantis isolates. Multi-locus sequence typing (MLST) and single nucleotide variant/polymorphism (SNP) SNVPhyl analysis showed that the *S*. Infantis isolates belong to sequence type ST32, likely share a common ancestor, and are closely related (1–3 SNP difference) to *bla*_CTX-M-65_-containing clinical and veterinary *S*. Infantis isolates from the United States and Latin America. Furthermore, phylogenetic analysis of SNPs following core-genome alignment (i.e., ParSNP) inferred close relatedness between the *S*. Infantis isolates from Galapagos and the United States. Prophage typing confirmed the close relationship among the Galapagos *S*. Infantis and was useful in distinguishing them from the United States isolates. This is the first report of MDR *bla*_CTX-M-65_-containing *S*. Infantis in the Galapagos Islands and highlights the need for increased monitoring and surveillance programs to determine prevalence, sources, and reservoirs of MDR pathogens.

## 1. Introduction

Non-typhoidal *Salmonella* (NTS) comprises multiple serovars of *Salmonella enterica* that can cause self-limiting or invasive enteric disease and are transmitted to humans mainly through contaminated food [1,2]. The consumption of poultry products represents a common route of NTS transmission to humans [3], and the increasing prevalence of antimicrobial resistance (AMR) among NTS isolates has become a serious concern [4,5]. Most gastrointestinal infections caused by NTS are self-limiting; however, complicated infections can be treated by first-line antibiotics such as ampicillin, folic pathway inhibitors, and chloramphenicol. Patients who are infected by multi-drug-resistant (MDR) NTS may require fluoroquinolones, third-generation cephalosporins, or monobactams to resolve the infection [1].

Antibiotic resistance is mediated by mutations in genes that are chromosomally encoded, or by genes carried by mobile genetic elements (MGE) such as plasmids, integrons, and transposons that are acquired from other bacteria in the environment through horizontal gene transfer [6]. Strains of AMR NTS are globally disseminated, and their emergence has been linked to the overuse of antibiotics in agriculture and human medicine [7]. European countries have banned the sub-therapeutic use of antibiotics as growth promoters in commercially farmed animals [8], and some member states have since reported a reduction in the prevalence of AMR bacterial pathogens in food animals [9]. However, the practice continues in many developing, and some developed countries, serving as potential reservoirs from which MDR strains of NTS emerge as a result of a sustained selection pressure [9,10].

In some Latin American countries, antibiotics are routinely fed to commercially grown and backyard chickens as growth promoters [11,12]. Unsurprisingly, the prevalence of AMR NTS in live poultry and poultry products from countries such as Colombia [13], Brazil [14,15], and Ecuador [16,17] is high. Extended-spectrum β-lactamase-producing (ESBL) strains of NTS are resistant to extended-spectrum cephalosporins and are frequently isolated from poultry sources in Latin America [16,18,19]. The *bla*_CTX-M_ gene family codes for a number of β-lactamase type enzymes that confer ESBL-producing properties to NTS and are commonly found in poultry-associated isolates from developing regions such as Latin America [18,20,21,22]. In Ecuador, *S.* Infantis is the most prevalent NTS associated with poultry, and *bla*_CTX-M-65_ is frequently detected in ESBL-producing isolates from this country [17,23]. The propensity of *bla*_CTX-M-65_-positive *S.* Infantis isolates to globally disseminate was highlighted by Brown et al., who showed a strong clonal relationship between strains detected in patients who traveled back to the United States from Peru and Ecuador, and other strains from Peru [24]. Additionally, previous work by Tate et al. revealed that *bla*_CTX-M-65_ was carried on a plasmid harbored by genetically similar *S.* Infantis isolates originating from food and patients in the United States and a patient in Italy [25].

The Galapagos Islands are an isolated territory of Ecuador that practices commercial poultry production and receives all inputs (baby chicks, feed, and medication) from the Ecuadorian mainland [26]. Some prevalence studies have reported the presence of drug-susceptible NTS isolates in wild animals on the Galapagos Islands [27,28,29]. Unlike in mainland Ecuador, where considerable information is available on the prevalence and AMR status of NTS in commercially grown poultry [17], similar work has not yet been reported for the Galapagos territory. The aim of this study was to genotypically and phenotypically characterize NTS isolated from poultry farms in the Galapagos Islands, using a WGS and antibiotic minimum-inhibitory concentration (MIC) approach.

## 2. Results

### 2.1. Determination of Serotype, AMR Phenotype, and Genotype

A total of seven NTS isolates, one per farm, was recovered from the 22 sampled farms. Serotyping and WGS analysis determined that two isolates (G10A and G11A) belonged to serotype *S*. Schwarzengrund. The remaining five isolates (G3A, G12A, G13A, G15A, and G17A) were typed as *S.* Infantis. All *S.* Infantis isolates exhibited MDR phenotypes (Table 1). All the isolates were susceptible to colistin (CL), azithromycin (AZM), tigecycline (TGC), and meropenem (MEM), (data not shown). One *S.* Infantis isolate (G17A) could not be recuperated for WGS analysis and characterization.

**Figure 1:**
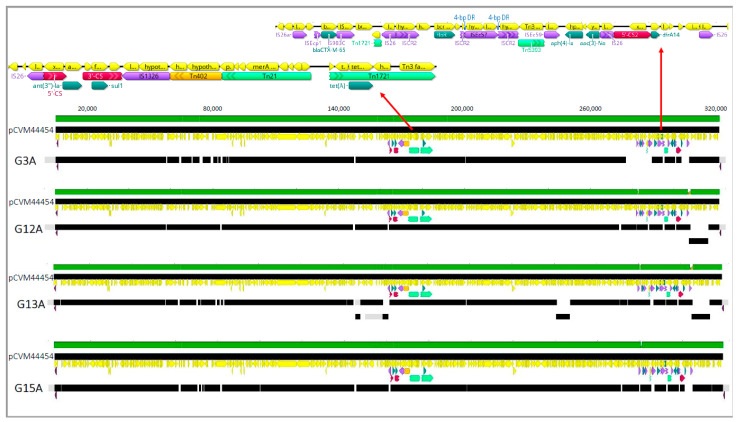
Plasmid map for isolates of *Salmonella* Infantis obtained from Galapagos Island containing antimicrobial. Diagram created in Geneious Prime. Assembled contigs from each sequenced isolate were mapped to the closed reference plasmid pCVM44454 (GenBank Accession CP016413), annotated using prokka (V1.14.0) and Galileo AMR (1, https://galileoamr.arcbio.com/mara/ accessed on 5 March 2021). Green bars show identity between reference plasmid (above) and mapped contigs (black bars) from each sequenced isolate. Two resistance regions are shown as larger images above. Resistance genes and cassettes are labeled and shown as teal arrows, conserved segments of integrons (5′-CS and 3′-CS) as pink arrows, insertion sequences as purple arrows, transposons as light green boxes, and direct repeats by blue labels. Position numbering of the resistance regions in the reference plasmid is shown above.

Genomic analysis revealed that the *S.* Schwarzengrund isolates lacked resistance genes against most of the antibiotics tested in our panel and displayed the corresponding drug-sensitive phenotypes (Table 2). The *qnrB19* gene was present in both *S.* Schwarzengrund isolates; however, only G10A exhibited reduced susceptibility to ciprofloxacin (CIP) (Table 2). All *S.* Infantis isolates possessed mutation D87Y in the *gyrA* gene conferring decreased susceptibility to CIP. *S*. Infantis isolates possessing tetracycline (*tetA*), trimethoprim (*dfrA14*), and sulfonamide (*sul1*) resistance genes displayed the corresponding resistance phenotypes. Similarly, multiple aminoglycoside resistance genes, *aph(4)-la*, *aadA1*, *aac(3)-IVa*, and *aph(3′)-Ia*, were detected in the *S.* Infantis genomes and corresponded to gentamicin (GEN) resistant phenotypes. Three *S.* Infantis isolates (G12A, G13A, and G15A) possessed the ESBL-producing *bla*_CTX-M-65_ gene and contigs mapped closely to the 316,160-bp IncFIB-like plasmid pCVM44454 (>298,000 identical bp), including the resistance region (Figure 1). Plasmid replicons belonging to type Col440II were detected in the genomes of both *S.* Schwarzengrund isolates.

### 2.2. MLST and SNP Analysis

Seven-gene (7-gene) MLST analysis revealed that the *S*. Schwarzengrund isolates (G10A and G11A) clustered in ST96, while all *S*. Infantis isolates clustered as ST32 (Table 2). We then used SNVPhyl to perform SNP-based phylogenetic analysis on the four *S*. Infantis isolates from Galapagos and ten previously reported *S*. Infantis isolates from the United States that were shown to carry *bla*_CTX-M-65_. The results indicated that the *S*. Infantis isolates from Galapagos were highly related to each other (0 pairwise SNP differences) and were also closely related to the *S*. Infantis isolates from the United States (1–3 pairwise SNP differences; Supplementary Table S1). Five of the United States isolates were highly related (0 pairwise SNP differences), while the remainder were as distantly related to each other, and to the other United States isolates (2–5 pairwise SNP differences), as they were from the Galapagos *S*. Infantis isolates.

**Figure 2:**
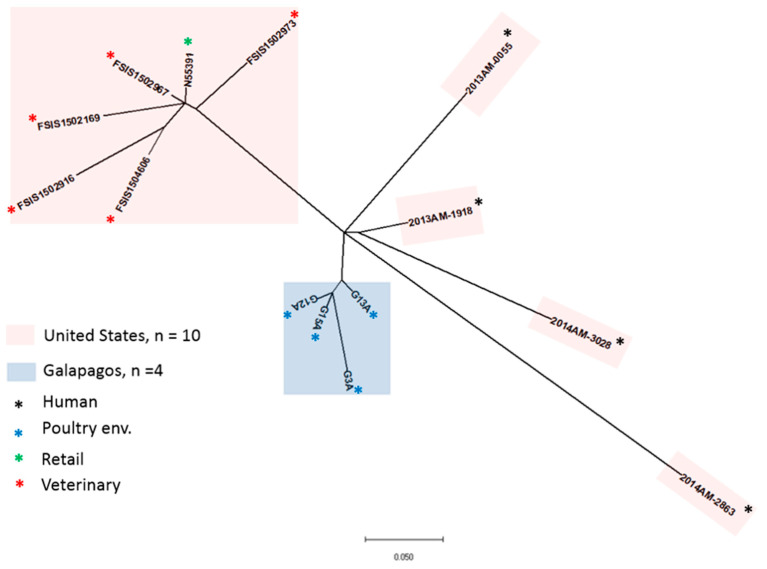
Phylogenetic analysis of *Salmonella* Infantis isolates from the Galapagos and the United States using single nucleotide polymorphism. Relationship among *S.* Infantis isolates from the Galapagos (*n* = 4) and the United States (*n* = 10; [25]) were analyzed by ParSNP analysis to identify single-nucleotide changes following a rapid core-genome multi-alignment of the genome sequences.

A ParSNP analysis focusing on the *S*. Infantis isolates from the Galapagos and the United States showed a more distinct branching of the Galapagos isolates. The United States isolates split into two groups (veterinary/retail and human), of which the veterinary/retail clustered together, while the remaining human isolates exhibited varying degrees of uniqueness but were phylogenetically closer to the Galapagos isolates (Figure 2).

### 2.3. Prophage Analysis

**Figure 3:**
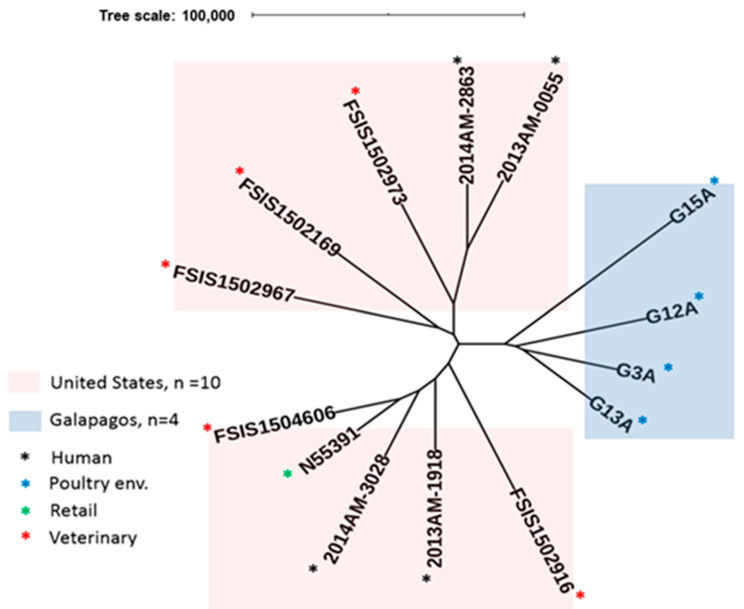
Phylogenetic analysis of *Salmonella* Infantis isolates from the Galapagos and the United States based on prophage sequence typing. Prophage sequences were extracted from the genome sequences of S. Infantis isolates from the Galapagos (n = 4) and the United States (n = 10; [25]).

Highly discriminatory prophage sequence typing further distinguished among the Galapagos isolates while preserving major branching patterns previously reported by Tate et al. for related *S*. Infantis isolates (Figure 3; Supplementary Table S2) [25]. The distinguishing features of each isolate, regardless of location, were demonstrable by the numbers and types of prophages present. Each of the *S. schwarzengurd* isolates possessed four prophages, but they all differed either in identity (1 prophage) or sizes of the prophage genomes (three phages). On the other hand, the four *S.* Infantis isolates had 5–7 prophages with distinct features, whereas the United States isolates reported by Tate et al. had 8–10 prophages [25].

## 3. Discussion

### 3.1. Potentially Pathogenic Clones of NTS Are Present in Poultry Farms in the Galapagos

There is some evidence that the global prevalence of AMR NTS isolates is partially driven by the overuse of antibiotics in human and veterinary medicine, as well as the movement of humans, animals and food commodities between different regions [30,31,32,33]. In this study, we identified MDR clones of *S.* Infantis in the Galapagos Island of Santa Cruz that are genetically similar to strains isolated from the United States and Ecuador. Here, we speculate that the importation of live poultry from Ecuador for commercial farming is a potential route for the entry of MDR *S*. Infantis into the Galapagos Islands. Furthermore, our study highlights the role of a large IncFIB-like plasmid in the global dissemination of ESBL-producing and MDR strains of *S.* Infantis, through poultry production.

Despite the apparently low recovery of *Salmonella* in the sampled poultry farm environments, both recovered serotypes (*S.* Schwarzengrund and *S.* Infantis) are known human pathogens. Interestingly, neither serotype was detected in previous surveillance studies of wildlife in several Galapagos Islands, including Santa Cruz [27,28,29]. As previously highlighted, poultry production in the Galapagos Islands receives all inputs, including day-old chicks, from Ecuador—a country in which *S.* Infantis has been reported to have a prevalence rate of ~42% in broiler chicken farms [16]. In fact, *S.* Infantis was the most abundant serotype contaminating chicken carcasses destined for retail in Ecuador [17,34]. In contrast, *S.* Schwarzengrund does not appear to be highly prevalent in Ecuador, but has been isolated from poultry and multiple food sources in Brazil [18,35,36] and Argentina [37]. To our knowledge, this is the first report on the occurrence of NTS in poultry from the Galapagos Islands.

WGS revealed that the *S.* Infantis isolates from this study were closely related and likely share a common ancestor. All isolates clustered into ST32, a globally disseminated clone that is highly prevalent in poultry and has been associated with diarrheal disease in affected humans [38]. Likewise, ST96 isolates of *S.* Schwarzengrund, also detected in this study, have been isolated from poultry-related products and the associated environments in Latin America and other countries [39,40,41]. Interestingly, SNP analyses showed a close relationship between *S.* Infantis isolates from the Galapagos and previously reported isolates originating from food sources in the US [25]. Additional resolution of the accessory genome using PST further revealed distinguishing features in each *S*. Infantis isolates. The high resolution provided by PST can be exploited to track the Galapagos isolates back to their potential place of origin in Ecuador and may prove to be an important laboratory support for future epidemiological investigations of the serovar *S*. Infantis (Table S2). We observed that the genome of *Salmonella* phage SJ46 was only present in the *S*. Infantis isolates from the United States, whereas the *Salmonella* phage g431c was present in both groups of *S*. Infantis isolates but not in the *S.* Schwarzengrund isolates. Three of the Galapagos *S*. Infantis had unique prophage sequences (*Bacillus* phage phi STI in G13A, *Clostridium* phage phi CTC2A in G3A and *Escherichia* phage pro 483 in G15A). Similarly, phage Entero P4 was unique to G12A among the Galapagos isolates. However, the majority of the United States isolates also had this phage.

### 3.2. NTS Isolates from the Galapagos Exhibit a Reduced Quinolone Susceptibility Phenotype

The *qnrB19* gene was the only AMR determinant detected in the genomes of the *S.* Schwarzengrund isolates and encodes plasmid-mediated quinolone resistance (PMQR), conferring reduced susceptibility to quinolones/fluoroquinolones by protecting the bacterial gyrase from interactions with the antibiotic [42]. The Col440II-like plasmid was detected in the *S.* Schwarzengrund isolates; however, a recent study highlighted that a small pPAB19-4-like plasmid plays an important role in the dissemination of *qnrB19* throughout Chile [43]. Not surprisingly, NAL susceptibility was detected in both *qnrB19*-positive *S.* Schwarzengrund isolates from this study, and one displayed an intermediate CIP phenotype. The ability of *qnrB19* to confer reduced quinolone/fluoroquinolone susceptibility in NTS of human and animal origin is well established [44,45,46]. Full resistance is usually exhibited by isolates that bear multiple quinolone resistance genes, including mutations within the quinolone-resistance-determining regions (QRDR) of the *gyrA*, *gyrB*, *parC* and *parE* genes [47]. Nonetheless, NAL and CIP-resistant *S.* Schwarzengrund isolates that possess *qnrB19*, but lack QRDR mutations, have been isolated from chicken by-products in Brazil [48].

The *S.* Infantis isolates in this study were all resistant to NAL but displayed intermediate MIC to CIP. Although these isolates lacked *qnrB19*, they possessed the D87Y mutation in the chromosomally encoded GyrA enzyme. Quinolones/fluoroquinolones target the bacterial DNA gyrase and topoisomerase enzymes, and mutations within the QRDR of *gyrA* can reduce their binding affinity for the antibiotics, rendering them ineffective [49]. Single-amino acid *gyrA* mutants display reduced quinolone susceptibility in multiple NTS serotypes, and full resistance has been observed in double-mutants [50,51] and strains that possess an additional PMQR such as *qnrB* [47]. All isolates from this study possessed the D87Y mutation commonly observed in quinolone-resistant strains of NTS [49]. The contribution of QRDR mutations to the rise of reduced quinolone/fluoroquinolone susceptibility and resistance in Latin America is well documented. For example, a surveillance study highlighted that the prevalence of quinolone-resistant NTS isolates, half of which possessed the D87N mutation in *gyrA*, was twice as high in Latin America (~14%) compared to North America (6.1%) [52].

MIC testing demonstrated that all *S.* Infantis isolates from the Galapagos Islands are multi-drug resistant. This feature has been reported in other studies in Ecuador, where most *S.* Infantis isolates presented multi-resistant phenotypes [16,17]. Moreover, resistance to third-generation cephalosporines mediated by the *bla*_CTX-M-65_ gene has been reported in Latin American countries, the United States and Europe [22,53,54]. Recently, this phenotype was reportedly associated with travel to South America [24], but more research is needed to ascertain whether *S.* Infantis strains carrying the *bla*_CTX-M-65_ gene are now endemic in countries where they are being increasingly detected.

### 3.3. S. Infantis Isolates Possess IncFIB-Like Plasmids That Encode for ESBL Production

ESBL-producing bacteria are characterized by resistance to AMP, extended-spectrum third-generation cephalosporins and monobactams [55]. Three *S*. Infantis isolates from this study possessed the *bla*_CTX-M-65_ gene that encodes a CTX-M β-lactamase. These enzymes constitute the most globally disseminated ESBL subgroup found in Gram-negative bacteria [56,57,58,59] and are often present in bacteria that contaminate live poultry and poultry by-products [60]. We detected the *bla*_CTX-M-65_ gene in a resistance region that was genetically similar to that in the IncFIB-like plasmid identified in ESBL-producing strains of *S*. Infantis (Figure 1) isolated from chicken by-products and patients in the US and Italy [25].

*Escherichia coli* and *Salmonella* isolates that contain *bla*_CTX-M_ genes have been reported in Latin American countries including Brazil and Argentina [61]. Furthermore, *bla*_CTX-M-65_-containing and ESBL-producing strains of *S.* Infantis have been isolated from poultry in Ecuador [16,62]. The use of antibiotics as prophylactics during the first week of life of chicks is a common practice in poultry production on Ecuador’s mainland; however, this is rarely practiced in the poultry industry of the Galapagos. It is noteworthy that cephalosporin antibiotics such as ceftiofur are often injected into fertile eggs at hatcheries to prevent *E.coli*-induced omphalitis in day-old chicks [63]. Moreover, a Canadian study revealed a strong correlation between this practice and the rise of ceftiofur-resistant strains of *Salmonella* Heidelberg [64]. In addition, Dierikx et al. demonstrated that the prevalence of ESBL-producing *E. coli* present in the poultry environment increased significantly after the use of β-lactam antibiotics [65]. The CTX-M family of ESBL enzymes are highly prevalent in *E. coli* [59], and the presence of *E. coli* strains that possess *bla*_CTX-M-65_ in Ecuadorian broilers chickens [66] highlights a potential source for the horizontal transfer of the gene to poultry-associated *S.* Infantis isolates in Ecuador. ESBL-producing strains of *S.* Infantis not only occupy specific niches on the Latin American mainland but can also disseminate to other localities. This was demonstrated when MDR clones that contained *bla*_CTX-M-65_ were isolated from foods and patients in the United States and shown to be closely related to an outbreak strain in Peru and Ecuador [24,25].

Taken together, the data from this study suggest that MDR strains of *S.* Infantis could potentially be transmitted from Ecuador to the Galapagos Islands through the movement of poultry-related inputs including day-old chicks, feed, personnel and other supplies between both places. This observation has significant implications from a public health standpoint, owing to the potential risk of transmission to humans and wildlife, and the potential difficulty in treating MDR infections. Indeed, suggestions have been made to construct hatcheries on the Galapagos Islands and to replace imported day-old chicks with locally available chickens [26]. This would reduce the reliance on externally sourced inputs, thus limiting potential incoming MDR NTS. However, further research is needed to pinpoint the source of MDR NTS in the Galapagos, and some of the tools described in this communication appear adequate to shed light on this need and thus provide optimism that measures can be developed and instituted to stem MDR NTS propagation in the Galapagos and elsewhere.

## 4. Materials and Methods

### 4.1. Sample Collection

Twenty-two broiler chicken farms stocked with the Cobb 500 breed, located on the island of Santa Cruz, which represented 54% of the broiler chicken farms in the Galapagos (n = 41), were sampled from February 2016 to April 2017. All farms were privately owned and reared between 6000 to 10,000 broiler chickens. Chickens were fed with compound feed prepared in Ecuador’s mainland. No growth promotors nor antibiotics were used during the rearing period, and chickens were between 35 to 42 days of age at the time of sampling. Farms were sampled once, resulting in the collection of 22 samples during the study period. Sampling was performed by walking twice inside and along the entire length of one barn per farm while wearing sterilized disposable overshoes. The used overshoes were then aseptically removed and stored in sterile bags on ice, then sent to a laboratory in Quito (Ecuador) within 12 h. *Salmonella* was isolated from the samples using the ISO 6579-1:2007 protocol [67]. All isolates were serotyped according to the Kauffmann–White scheme [68].

### 4.2. Resistance Phenotyping

The AMR phenotype of all confirmed NTS isolates was determined by evaluating MIC values with broth microdilution EUVSEC plates (Thermo Scientific, West Palm Beach, USA) according to the manufacturer instructions. The following antibiotics were evaluated: sulfamethoxazole (SMX), trimethoprim (TMP), gentamicin (GEN), ciprofloxacin (CIP), nalidixic acid (NAL), ampicillin (AMP), cefotaxime (CTX), ceftazidime (TAZ), tetracycline (TET), chloramphenicol (CHL), colistin (CL), azithromycin (AZM), tigecycline (TGC) and meropenem (MEM). *E. coli* ATCC 25922 was used as the quality control strain.

Epidemiological cutoff values (ECOFF) derived from EUCAST were used to determine the presence and level of phenotypic resistance in the *Salmonella* isolates [69]. For those antibiotics for which ECOFF values are not published (SMX, CL and AZM), clinical breakpoint values from the Clinical and Laboratory Standards Institute (CLSI) or previously recommended criteria were used [70]. All intermediate phenotypes obtained with breakpoint values from CLSI were considered as resistant since they are expected to harbor genetic determinants of antimicrobial resistance. Multi-resistant isolates were defined as those having resistance to three or more antibiotics.

### 4.3. DNA Extraction and Whole-Genome Sequencing

Genomic DNA was extracted and purified using Qiagen DNeasy Blood & Tissue Kit (Qiagen Sciences Inc., Germantown, Frederick, MD, USA). DNA concentrations were measured using the Qubit^®^ Fluorimeter for quantification of double-stranded DNA and the Qubit dsDNA BR Assay kit (Invitrogen/Thermo Fisher Scientific, Waltham, MA, USA). Additional quality assessments were made using the NanoDrop 2000 UV-Vis (Thermo Fisher Scientific, Waltham, MA, USA) for determination of A_260/280_ values. Whole-genome sequencing (WGS) was performed using the MiSeq platform (Illumina, San Diego, CA, USA) according to the harmonized FDA GenomeTrakr/CDC PulseNet protocol (https://www.cdc.gov/pulsenet/pathogens/protocols.html; last accessed on 8 January 2018).

### 4.4. Bioinformatic Analyses

Raw reads were submitted to GenomeTrakr and assembled de novo using their internal analysis pipeline. Genome assemblies were submitted to EnteroBase (https://enterobase.warwick.ac.uk/species/index/senterica; last accessed on 15 January 2020) in order to determine the 7-MLST profile of each isolate based on the Acthman scheme and confirm serotype designation using the SISTR algorithm [71]; https://lfz.corefacility.ca/sistr-app/; last accessed on 16 January 2020. Antimicrobial resistance genes were identified using the ResFinder database (https://bitbucket.org/genomicepidemiology/resfinder_db; last accessed on 17 January 2020, 90% ID and 60% gene coverage cutoffs). For mutational resistance, *gyrA* and *parC* sequences were extracted from genome assemblies using a custom Perl script and aligned to identify mutations. For plasmid analysis, the assembled contigs from each sequenced isolate were annotated using Prokka (V1.14.0) and Galileo AMR (https://galileoamr.arcbio.com/mara/; last accessed on 30 January 2020) and mapped to the reference plasmid pCVM44454 (GenBank Accession CP016413), isolated from clinical *S.* Infantis from the US [25]. Single-nucleotide variations among the *S.* Infantis Galapagos isolates were examined using two separate tools, namely SNVPhyl [72] and ParSNP [73] and compared to another ten isolates of *S.* Infantis obtained from the US as previously reported [25]. A fully assembled *S.* Infantis genome (4,710,675 bp, LN649235) was used as a reference for the SNVPhyl analysis, while the auto-recruit option was used for the choice of the reference genome for ParSNP analysis. The tools provided different degrees of stringency for evaluating relatedness among the different isolates. To further evaluate the relationship among the isolates, we used a highly discriminatory phage sequence typing (PST) tool [74], capable of exploring changes in the mobile accessory genome of *Salmonella* isolates, which usually contains prophages [75]. We identified the presence of prophage sequences in the genome of each strain using PHASTER (https://phaster.ca/; last accessed on 3 February 2020) and clustered related sequences CD-HIT-EST (http://weizhongli-lab.org/cd-hit/; last accessed on 3 February 2020) with sequence identity and length parameter cut off set at 99%, and the relationships were displayed as a phylogenetic tree by means of QIIME software (https://qiime2.org/; last accessed on 4 February 2020).

## 5. Conclusions

The present study highlights the global dissemination of poultry-associated isolates of MDR NTS, even in an area that prohibits the non-therapeutic use of antibiotics in poultry farming. The continuing spread of resistance to ESBL and fluoroquinolones, which represents two important groups of antibiotics for human use, remains a cause of concern. Careful analyses to track AMR spread are needed using informative tools that can shed adequate light needed to mount effective control measures. Discriminatory phage-based analysis can provide useful insight for understanding the epidemiology and spread of MDR NTS isolates.

## Figures and Tables

**Table 1:** Minimum Inhibitory Concentration values (μg/mL) for various antibiotics against strains of *Salmonella* Infantis and *Salmonella* Schwarzengrund.

Isolate	Serotype	SMX	GEN	CIP	AMP	CTX	TAZ	TET	TMP	CHL	NAL
G3A	Infantis	(512)	(8)	(0.25)	1	0.25	0.5	(64)	(32)	(128)	(128)
G12A	Infantis	(1024)	(8)	(0.12)	(64)	(4)	(4)	(64)	(32)	(64)	(128)
G13A	Infantis	(1024)	(8)	(0.12)	(64)	(4)	(4)	(64)	(32)	(128)	(64)
G15A	Infantis	(1024)	(8)	(0.25)	(64)	(4)	(4)	(64)	(32)	(128)	(128)
G10A	Schwarzengrund	64	1	(0.50)	1	0.25	0.5	2	0.25	8	16
G11A	Schwarzengrund	64	2	0.02	1	0.25	0.5	2	0.25	8	4

Numbers in parenthesis indicate resistant phenotypes. All isolates were susceptible to colistin, azithromycin, tigecycline, and meropenem (data not shown).

**Table 2:** Genetic typing and determinants of antimicrobial resistance genes in *Salmonella* isolates from Galapagos Islands.

Isolate	MLST	Plasmid	Β-Lactam	Quinolone	Tetracycline	Trimethoprim	Sulfonamide	Aminoglycoside
G3A	ST-32	IncFIB-like	-	*gyrA* D87Y	*tet(A)*	*dfrA14*	*sul1*	*aph(4)-la, aadA1, aac(3)-IVa, aph(3′)-Ia*
G12A	ST-32	IncFIB-like	*bla*_CTX-M-65_	*gyrA* D87Y	*tet(A)*	*dfrA14*	*sul1*	*aph(4)-la, aadA1, aac(3)-IVa, aph(3′)-Ia*
G13A	ST-32	IncFIB-like	*bla*_CTX-M-65_	*gyrA* D87Y	*tet(A)*	*dfrA14*	*sul1*	*aph(4)-la, aadA1, aac(3)-IVa, aph(3′)-Ia*
G15A	ST-32	IncFIB-like	*bla*_CTX-M-65_	*gyrA* D87Y	*tet(A)*	*dfrA14*	*sul1*	*aph(4)-la, aadA1, aac(3)-IVa, aph(3′)-Ia*
G10A	ST-96	Col440II	-	*qnrB19*	*-*	*-*	*-*	*-*
G11A	ST-96	Col440II	-	*qnrB19*	*-*	*-*	*-*	*-*

## Data Availability

Genome sequences of all isolates sequenced as part of this study have been deposited in the GenBank under BioProject Number PRJNA377900.

## Supplementary Materials

The following are available online at https://www.mdpi.com/2079-6382/10/3/267/s1. Supplementary Table S1: Matrix of single nucleotide differences between isolates of *Salmonella enterica* serovar Infantis and *Salmonella enterica* serovar Schwarzengrund; Supplementary Table S2: Prophages in the genomes of *Salmonella enterica* serovars Infantis and *Salmonella enterica* serovar Schwarzengrund.
