# Supplementary material for: Whole-Genome Sequencing Reveals the Presence of the blaCTX-M-65 Gene in Extended-Spectrum β-Lactamase-Producing and Multi-Drug-Resistant Clones of Salmonella Serovar Infantis Isolated from Broiler Chicken Environments in the Galapagos Islands
Source: Antibiotics (Basel). 2021 Mar 5;10(3):267. doi: 10.3390/antibiotics10030267 (PMC8000398; doi:10.3390/antibiotics10030267)
Supplement: Supplementary file 1 [file antibiotics-10-00267-s001.pdf]

## Supplementary Materials

**Table S1.** Matrix of single nucleotide differences among isolates of *Salmonella enterica* serovar *infantis.*

| **Strain** | **2014AM-3028** | **2014AM-2863** | **2013AM-1918** | **N55391** | **FSIS 1504606** | **FSIS 1502973** | **FSIS 1502169** | **FSIS 1502967** | **FSIS 1502916** | **2013AM-0055** | **G12A** | **G13A** | **G15A** | **G3A** |
|---|---|---|---|---|---|---|---|---|---|---|---|---|---|---|
| 2014AM-3028 | - | | | | | | | | | | | | | |
| 2014AM-2863 | 5 | - | | | | | | | | | | | | |
| 2013AM-1918 | 3 | 4 | - | | | | | | | | | | | |
| N55391 | 4 | 3 | 3 | - | | | | | | | | | | |
| FSIS1504606 | 4 | 3 | 3 | 0 | - | | | | | | | | | |
| FSIS1502973 | 4 | 3 | 3 | 0 | 0 | - | | | | | | | | |
| FSIS1502169 | 4 | 3 | 3 | 0 | 0 | 0 | - | | | | | | | |
| FSIS1502967 | 4 | 3 | 3 | 0 | 0 | 0 | 0 | - | | | | | | |
| FSIS1502916 | 4 | 3 | 3 | 0 | 0 | 0 | 0 | 0 | - | | | | | |
| 2013AM-0055 | 4 | 3 | 3 | 2 | 2 | 2 | 2 | 2 | 2 | - | | | | |
| G12A | 3 | 2 | 2 | 1 | 1 | 1 | 1 | 1 | 1 | 1 | - | | | |
| G13A | 3 | 2 | 2 | 1 | 1 | 1 | 1 | 1 | 1 | 1 | 0 | - | | |
| G15A | 3 | 2 | 2 | 1 | 1 | 1 | 1 | 1 | 1 | 1 | 0 | 0 | - | |
| G3A | 3 | 2 | 2 | 1 | 1 | 1 | 1 | 1 | 1 | 1 | 0 | 0 | 0 | - |
| Reference | 14 | 13 | 13 | 12 | 12 | 12 | 12 | 12 | 12 | 12 | 11 | 11 | 11 | 11 |

Comparison of single nucleotide variants among genome sequences belonging to *Salmonella enterica* serovar Infantis (n=10) obtained from the Galapagos Island (G3A, G11A, G13A and G15A) and the United States was carried out using SNVPhyl algorithm and *Salmonella enterica* serovar Infantis strain LN649235 as the reference genome. Number of different variants between genomes are tabulated.

**Table S2.** Prophages in the genomes of *Salmonella enterica* serovars *Infantis* and *S. e. serovar*.

| | **Size of prophage genome (Kb)** | | | | | | | | | | | | | | | |
|---|---|---|---|---|---|---|---|---|---|---|---|---|---|---|---|---|
| **Location** | **Galapagos** | | | | | | United States | | | | | | | | | |
| **Serovar** | **Schwarzengrund** | | | | | | **Infantis** | | | | | | | | | |
| **Isolate identity:** | **G10A** | **G11A** | **G3A** | **G12A** | **G13A** | **G15A** | **2014AM-2863** | **FSIS 1504606** | **FSIS 1502973** | **FSIS1502967** | **FSIS 1502169** | **FSIS 1502916** | **2013AM-0055** | **2013AM-1918** | **N55391** | **2014AM-3028** |
| No of prophages: | 4 | 4 | 7 | 6 | 6 | 5 | 9 | 10 | 9 | 9 | 9 | 10 | 9 | 8 | 9 | 9 |
| Prophage genome identity | | | | | | | | | | | | | | | | |
| Phage Bacill phi NT1 | | | | | 12.8 | | | | | | | | | | | |
| Phage Burkho BcepMU | | | 18 | 19.5 | | 18 | 20.4 | 20.4 | 20.4 | 20.4 | 20.4 | 20.4 | 20.4 | 20.4 | 20.4 | 20.4 |
| Phage Clostr phiCTC2A | | | 10.3 | | | | | | | | | | | | | |
| Phage Edward GF | 24.2 | 49.6 | | | | | | | | | | | | | | |
| Phage Escher pro483 | | | | | | 31.5 | | | | | | | | | | |
| Phage Entero P4 | | | | 18.6 | | | 18.6 | 18.6 | 18.6 | | 18.6 | 18.6 | 18.6 | 18.6 | 18.6 | 10.4 |
| Phage Entero BP 4795 | | | 35.8 | 35.8 | | 35.8 | | 35.8 | 35.8 | 18.6 | | | 35.8 | 35.8 | | 35.8 |
| Phage Entero ES18 | 38.4 | 49.3 | | | | | | | | | | | | | | |
| Phage Entero mEp237 | 28.4 | | | | | | | | | | | | | | | |
| Phage Gifsy 1 | | | 9.1 | 9.1 | 13.8 | | 32 | 28.9 | 28.9 | 32, 9.6 | 32 | 28.9 | 32, 9.1 | 28.9, 9 | 28.9 | 28.9, 9 |
| Phage Salmon BTP1 | | | | | | | | 17.6 | | | | 17.6 | | | | |
| Phage Salmon g341c | | | 15.5 | 17.9 | 15.5 | 17.9 | 17.9 | 17.9 | 17.9 | 17.9 | 17.9 | 17.9 | 17.9 | 17.9 | 17.9 | 17.9 |
| Phage Salmon 118970 Sal4 | 29 | 29.2 | | | | | | | | | | | | | | |
| Phage Salmon Fels 1 | | 15.6 | | | | | | | | | | | | | | |
| Phage Salmon SJ46 | | | | | | | 5.1 | 5.1 | 5.1 | 7.8 | 5.1 | 7.8 | 7.8 | 5.1 | 5.1 | 7.8 |

| | | | | | | | | | | | | | | |
|---|---|---|---|---|---|---|---|---|---|---|---|---|---|---|
| Phage Shigel POCJ13 | 12.8 | | | | 12.8 | | 12.8 | 12.8 | | 12.8 | | 12.8 | | 12.8 |
| Phage Shigel Stx | | | | | | | | | 12.8 | | | | | |
| Phage Sodali phi SG1 | | | | | | 12.8 | | | | | 12.8 | | 12.8 | |
| Phage Strept Sfi 19 | | | 20.1 | 20.1 | 9.1 | 9.1 | 9.1 | | 9.1 | 9 | | | 9.8 | |
| Phage Stx2 | | | 35.8 | | 35.8 | | | 35.8 | 35.8 | 35.8 | | | 35.8 | |
| Phage Yersin_L_413C | 25.2 | 25.2 | 25.2 | | 35.3 | 25.2 | 35.3 | 31.5 | 25.2 | 31.5 | 35.3 | | 25.2 | 25.2 |

Presence and size of prophage sequences present in the genomes of *Salmonella enterica* serovar Infantis (*n* = 10) obtained from the Galapagos Island (G3A, G12A, G13A.and G15A) and the United States, and S. e. serovar Schwarzengrund (*n* = 2; G10A and G11A) from the Galapagos Islands were determined using the PHASTER algorithm.
